# Metabolite-Specific Echo-Planar Imaging of Hyperpolarized [1-^13^C]Pyruvate at 4.7 T

**DOI:** 10.3390/tomography7030040

**Published:** 2021-09-15

**Authors:** Tyler Blazey, Galen D Reed, Joel R Garbow, Cornelius von Morze

**Affiliations:** 1 Mallinckrodt Institute of Radiology, Washington University, St. Louis, MO 63110, USA; blazey@wustl.edu (T.B.); garbow@wustl.edu (J.R.G.); 2 GE Healthcare, Dallas, TX 75390, USA; galen.reed@ge.com

**Keywords:** dynamic nuclear polarization, molecular imaging, liver, EPI

## Abstract

Although hyperpolarization (HP) greatly increases the sensitivity of ^13^C MR, the usefulness of HP in vivo is limited by the short lifetime of HP agents. To address this limitation, we developed an echo-planar (EPI) sequence with spectral-spatial radiofrequency (SSRF) pulses for fast and efficient metabolite-specific imaging of HP [1-^13^C]pyruvate and [1-^13^C]lactate at 4.7 T. The spatial and spectral selectivity of each SSRF pulse was verified using simulations and phantom testing. EPI and CSI imaging of the rat abdomen were compared in the same rat after injecting HP [1-^13^C]pyruvate. A procedure was also developed to automatically set the SSRF excitation pulse frequencies based on real-time scanner feedback. The most significant results of this study are the demonstration that a greater spatial and temporal resolution is attainable by metabolite-specific EPI as compared with CSI, and the enhanced lifetime of the HP signal in EPI, which is attributable to the independent flip angle control between metabolites. Real-time center frequency adjustment was also highly effective for minimizing off-resonance effects. To the best of our knowledge, this is the first demonstration of metabolite-specific HP ^13^C EPI at 4.7 T. In conclusion, metabolite-specific EPI using SSRF pulses is an effective way to image HP [1-^13^C]pyruvate and [1-^13^C]lactate at 4.7 T.

## 1. Introduction

Carbon-13 magnetic resonance spectroscopy (^13^C MRS) can provide noninvasive access to specific elements of intermediary metabolism in vivo. However, applicability for medical imaging has been hampered by the low in vivo concentration and intrinsic sensitivity of ^13^C. Hyperpolarization can overcome these limitations by amplifying the polarization of ^13^C by up to five orders or magnitude, through the process of dynamic nuclear polarization (DNP) [1], or by other methods such as parahydrogen-induced polarization (PHIP) [2]. Hyperpolarization vastly increases the signal-to-noise ratio (SNR) of ^13^C MRS, making it possible to capture dynamic metabolic images in vivo [3].

The main limitation of hyperpolarized (HP) ^13^C MRS is the short lifetime of HP magnetization. Polarization is subject to rapid and irreversible spin–lattice (T_1_) relaxation, and therefore the most useful HP agents have long T_1_ relaxation times. To date, the most widely investigated HP agent is [1-^13^C]pyruvate [4], which has a T_1_ of about 65 s in aqueous solution at 4.7 T. As it can be used to monitor the conversion of pyruvate to lactate, [1-^13^C]pyruvate has primarily been used in the context of cancer imaging, as many tumors exhibit excessive aerobic glycolysis [5].

The short lifetime of hyperpolarized species demands rapid, efficient MR pulse sequences. In addition to T_1_ relaxation, polarization is also consumed by T_2_ relaxation following each RF excitation pulse. It is therefore advantageous to develop pulse sequences that conserve as much polarization as possible while still providing a sufficient SNR. Initial HP ^13^C metabolic imaging studies used chemical shift imaging (CSI) [3,6], which requires a separate RF excitation for each k-space location. This is less than ideal for HP imaging, since it requires many excitations. The slow CSI acquisition is also highly susceptible to motion-induced blurring as well as k-space filtering effects resulting from relaxation.

Accelerated pulse sequences that have been used successfully for HP metabolic imaging include echo-planar spectroscopic imaging (EPSI) [7,8] and echo-planar imaging (EPI) [9,10,11]. As with CSI, EPSI also acquires a ^13^C spectrum at each voxel but encodes an entire line of spatial k-space data per excitation, providing significant acceleration [12]. Alternatively, the EPI approach eschews the acquisition of localized spectra in favor of metabolite-specific imaging, with single-shot EPI acquiring the entire k-space trajectory with a single excitation [13]. This not only preserves polarization by reducing the number of RF pulses but can also greatly improve the temporal resolution.

Since spectral components are not easily resolved in EPI, it is advantageous to design RF excitations with both spatial and spectral selectivity [14]. Such excitation pulses are typically referred to as spectral-spatial RF (SSRF) pulses [15,16,17]. With a metabolite-specific SSRF pulse, each EPI acquisition provides an image of a single metabolite at a single point in time. Given the fast acquisition time of a typical EPI sequence (~100 ms per slice), images of multiple metabolites can be collected rapidly in succession at each time point. A significant further advantage of metabolite-specific excitation is that a different flip angle can be used for each metabolite. Typically, a smaller flip angle is used on the injected substrate (e.g., [1-^13^C]pyruvate) to preserve polarization for metabolic conversion to downstream metabolites [16]. A larger flip angle is then used for the polarized product (e.g., [1-^13^C]lactate), as it is present in smaller amounts than the substrate and therefore benefits from the increased SNR provided by a larger flip angle. Furthermore, the polarized product consumed by the RF pulse can be regenerated from the substrate metabolically.

The purpose of this study was to develop an EPI sequence for efficiently imaging HP [1-^13^C]pyruvate and its metabolic product [1-^13^C]lactate at 4.7 T. To our knowledge, this is the first report of HP ^13^C EPI at 4.7 T based on metabolite-specific SSRF excitation. To accomplish this, we designed custom SSRF excitation pulses for each metabolite and integrated them into a robust existing flyback EPI sequence. We also developed a pulse sequence for measuring SSRF profiles on our scanner platform (Agilent), which we share online along with the new pulse waveforms. In addition, we developed a fully automated procedure for determining and applying appropriate center frequency shifts for the ^13^C excitation pulses (code also shared), based on real-time scanner feedback to make our sequence more robust to off-resonance effects.

## 2. Materials and Methods

### 2.1. Sequence Development

**Figure 1:**
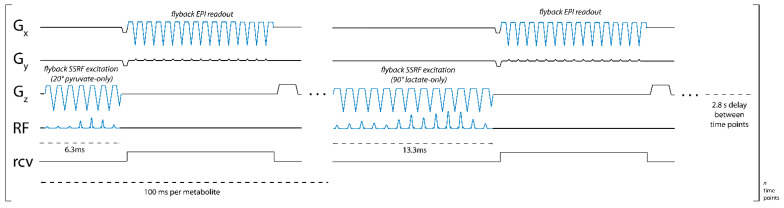
Pulse sequence diagram for metabolite-specific 2D EPI imaging of [1-^13^C]pyruvate and [1-^13^C]lactate at 4.7 T. An image of both pyruvate and lactate was acquired every 2.8 s, with a 100 ms acquisition time for each image.

**Figure 2:**
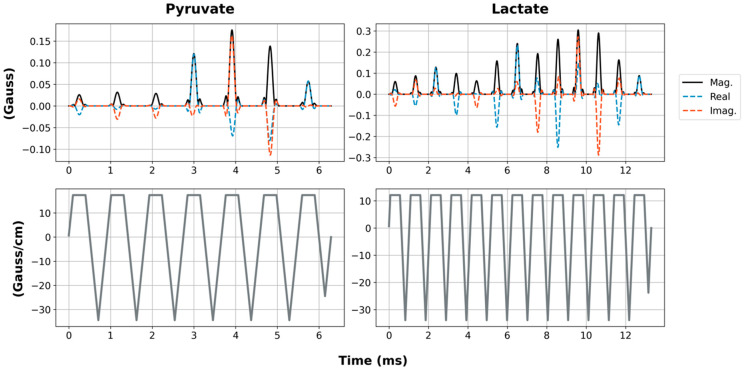
RF pulse waveforms for pyruvate and lactate SSRF pulses (**top** row). The solid black lines are the pulse magnitude, while the dashed blue and red lines are the real and imaginary components, respectively. The gradient waveforms are shown on the **bottom** row.

We developed a pulse sequence for metabolite-specific 2D EPI of HP [1-^13^C]pyruvate and its downstream metabolic product [1-^13^C]lactate at 4.7 T (50.7 MHz). As diagrammed in Figure 1, pyruvate and lactate images were acquired alternately, with a user-specified time delay between each rapid set of metabolite images. To accomplish this, we designed custom SSRF pulses to specifically excite either [1-^13^C]lactate or [1-^13^C]pyruvate signals at a given spatial location while maximally avoiding all other related species, including each metabolite’s redox partner as well as [1-^13^C]pyruvate-hydrate and [1-^13^C]alanine. For a more robust performance, SSRF excitation was performed using flyback gradient waveforms. The custom pulse waveforms are shown in Figure 2. SSRF pulse widths were 6.3 ms for pyruvate-only 20° excitation (B_1,peak_ = 0.2 G) and 13.3 ms for lactate-only 90° excitation (B_1,peak_ = 0.3 G). Each pulse was designed for uniform excitation over a ±0.5 ppm passband, with a time–bandwidth product of 3.5 and maximum ripple of 1%. Pulses were designed using the “Spectral-Spatial RF Pulse Design for MRI and MRSI” MATLAB package (https://github.com/LarsonLab/Spectral-Spatial-RF-Pulse-Design, accessed on 12 May 2021) [16] and integrated with a previously described EPI pulse sequence used for HP ^13^C MRI studies at 14 T [18]. As with the excitation pulses, flyback EPI readout waveforms were employed for a robust imaging performance without the need for odd/even gradient corrections.

We also developed a pulse sequence for quickly measuring SSRF excitation profiles (both spectral and spatial) on the Agilent MRI platform. This was accomplished by modifying a vendor-supplied 1D profile imaging sequence. Since several sequence changes were necessary to move the slice and readout gradients onto the same axis for pulse profile measurement, and no existing tools were available, we opted to share the source code for the modified SSRF profile sequence online (https://github.com/cvonmorze/SSRFprofileAgilent, accessed 15 May 2021). To maximize the SNR, all profile measurements were performed in ^1^H mode using a long cylindrical water phantom. For profile testing, the pulse center frequency was swept over a range of ±600 Hz, slightly exceeding the spectral bandwidth of both custom pulses. Resulting experimental profiles were compared to magnetization profiles computed in simulation. For comparison, the experimental profiles were interpolated to match the spatial and spectral range of the simulated profiles.

### 2.2. In Vivo Testing

For the in vivo testing, 100 mM HP [1-^13^C]pyruvate was generated in a GE 5T SPINlab hyperpolarizer and quickly transferred to a 4.7 T MRI scanner (Agilent, Santa Clara, CA, USA) equipped with a 72 mm i.d. dual-tuned ^13^C/^1^H volume coil (ExtendMR, Milpitas, CA, USA). The contrast materials were transferred to the MRI scanner located one floor below the polarizer through a sample drop chute installed adjacent to the polarizer while protected magnetically inside a handheld electromagnet carrier with an internal static field of 50 G [19]. The transfer time was ~30 s. Approximately 20 s after 2.5 mL of HP pyruvate was injected (~12 s injection) into the tail vein of an anesthetized (isoflurane) rat, images of pyruvate and lactate were acquired every 3 s for 60 s (20 images for each metabolite). Images were acquired coronally through the rat body, with a 16 × 16 matrix (80 × 80 mm^2^ FOV) for a 5 mm in-plane resolution and 20 mm slice thickness. To preserve HP magnetization for metabolic conversion over time, a lower flip angle was applied on the injected [1-^13^C]pyruvate substrate (20°) compared with the downstream [1-^13^C]lactate product (90°).

For comparison with a conventional spectroscopic imaging approach, we also performed 2D dynamic chemical shift imaging (CSI) following an identical injection of HP pyruvate during the same imaging session. Six dynamic CSI images with a temporal resolution of approximately 6 s were acquired starting 20 s after the start of injection. Imaging parameters for the CSI sequence were as follows: matrix = 8 × 8, spatial resolution = 10 × 10 mm^2^, FOV = 80 × 80 mm^2^, flip angle = 10° (hard pulse), spectral bandwidth = 25 kHz, spectral points = 2048, TR = 82 ms. For anatomic reference, a ^1^H gradient echo multislice (GEMS) image was also acquired (matrix = 128 × 128, FOV = 80 × 80 mm^2^).

During imaging, a Gd-doped aqueous [^13^C]urea vial phantom was placed on the posterior aspect of the rat’s abdomen. This phantom served two purposes. First, it allowed us to calibrate the power of the RF pulses. Based on hard pulse calibration, our 72 mm volume ^13^C coil provides a maximum B_1_ of ~2 G when mated with our scanner’s 1 kW low-band amplifier (Model 3200, American Microwave Technology, Anaheim, CA, USA), which must be scaled down for the SSRF excitations according to the peak B_1_ values quoted above. Second, for our initial experiments, we used the resonance frequency of the [^13^C]urea phantom in the CSI experiment as a reference for the frequency offsets used by the pyruvate-only and lactate-only SSRF excitation pulses. To ensure that the frequency offsets were accurate, we also used the CSI experiment to measure the in vivo resonance frequencies of HP pyruvate and lactate. Later, we developed a procedure that automatically determines and applies the correct frequency offsets in real time at the start of the HP imaging experiment.

For this automated procedure, following the injection of [1-^13^C]pyruvate, a single-slice selective ^13^C spectrum was acquired from a 4 cm-thick axial slice including the liver and kidneys but excluding the heart and lungs. A low 5° flip angle was used to preserve magnetization for subsequent EPI scans. Other relevant parameters were as follows: spectral bandwidth = 10 kHz, spectral points = 2048, TR = 1.2 s, and TE = 0.67 ms. Next, a custom script based on VNMRJ commands was triggered automatically to move the scanner center frequency to the pyruvate resonance and set the frequency offsets for both pulses relative to pyruvate. Finally, the custom script triggered the dynamic EPI acquisition.

Since the SSRF passbands were not centered on the resonances of interest, in-plane reconstruction shifts were applied to all EPI images to account for frequency offset between each metabolite and the center frequency of the pulse. These shifts were determined by the spectral distance between each pulse center frequency and the corresponding metabolite of interest, separately as a fraction of the imaging bandwidth along the frequency- and phase-encoding dimensions.

### 2.3. Imaging Processing

For CSI, an exponential apodization filter of 12 Hz was applied, and metabolite images were computed by taking the peak height of each resonance of interest. Image processing was performed using the Nmrglue [20], Numpy [21], Scipy [22], and TensorLy [23] packages in Python.

## 3. Results

**Figure 3:**
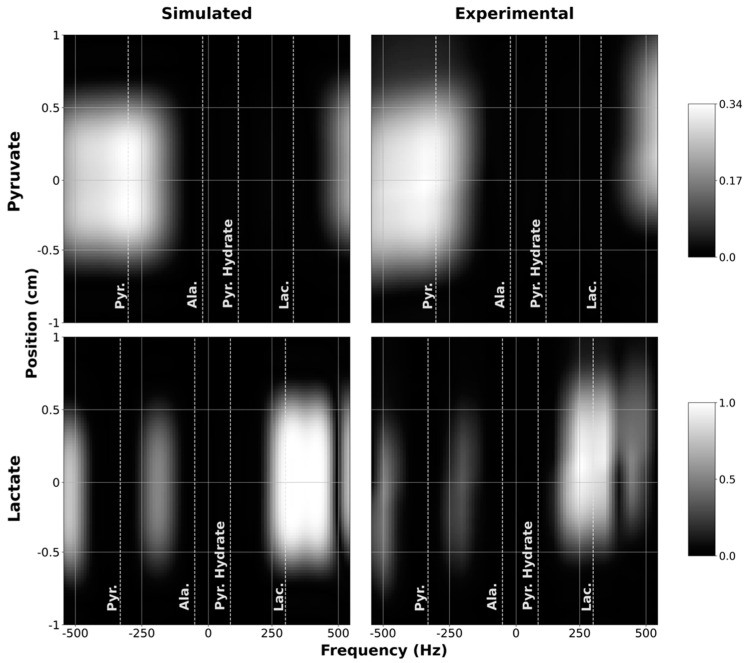
Simulated (1st column) and measured (2nd column) spectral-spatial profiles for pyruvate (1st row) and lactate (2nd row). Measured profiles were obtained by sweeping the scanner center frequency from −600 to +600 Hz. For display purposes, the measured profiles were interpolated onto the same grid points as the simulated profiles.

To verify that the custom SSRF pulses generate the expected spatial and spectral excitation profiles, we compared the simulated profiles of the resulting transverse magnetization as a function of space and frequency against experimental profiles measured inside the scanner. These results were obtained by scanning a long, narrow cylindrical water phantom using the customized 1D profile sequence in ^1^H mode. As depicted in Figure 3, good congruence was observed between the simulated and experimental magnetization profiles for both the pyruvate-only and lactate-only SSRF excitation pulses. The pyruvate passband effectively captures the pyruvate resonance (172.0 ppm) while excluding the resonances for lactate (184.3 ppm), alanine (177.7 ppm), and pyruvate hydrate (180.4 ppm). The narrower passband of the lactate-only excitation pulse likewise effectively excites lactate without exciting the other metabolites.

**Figure 4:**
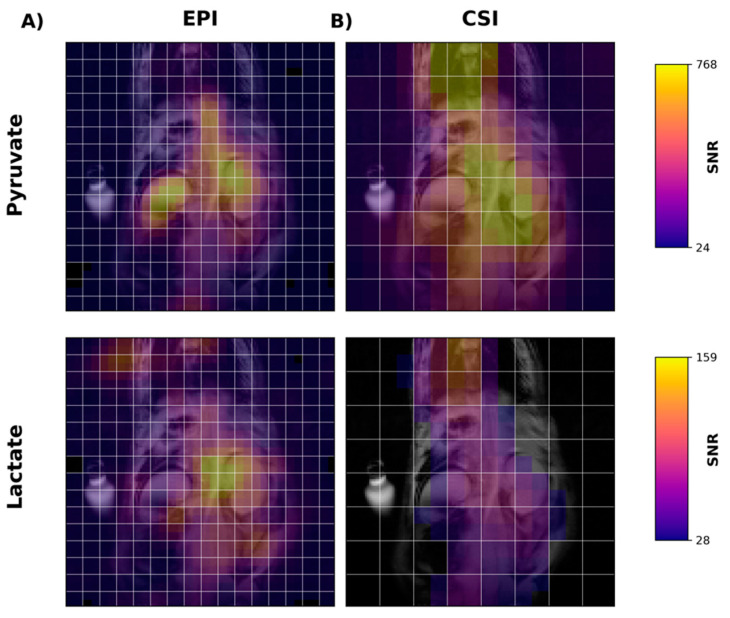
Metabolite images of [1-^13^C]pyruvate (1st row) and [1-^13^C]lactate (2nd row) in the liver and kidney of a rat following injection of [1-^13^C]pyruvate. Images in the first column (**A**) were acquired using metabolite-specific EPI (Figure 1), while images in the second column (**B**) were acquired using a traditional CSI sequence. All images were obtained by summing the signal over all time points. White lines are acquisition grids for each image. Images are shown after a 2× linear interpolation followed by nearest neighbor interpolation. EPI images required 4× nearest neighbor interpolation and CSI 8×. Color scale shows each metabolite in SNR units.

Next, we performed in vivo imaging of the rat abdomen following the injection of HP [1-^13^C]pyruvate. Figure 4A shows temporally summed pyruvate and lactate images obtained using the new metabolite-specific EPI sequence. For both metabolites, the HP signal is detectable in both the liver and kidney. For comparison and for confirmation of SSRF pulse center frequencies, we also obtained CSI images following HP [1-^13^C]pyruvate injection in the same rat (Figure 4B). CSI spectra from the liver and kidney are shown in Figure S1.

**Figure 5:**
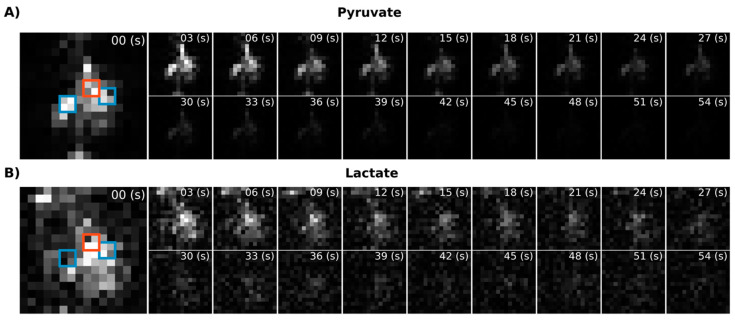
Dynamic metabolic images for [1-^13^C]pyruvate (**A**) and [1-^13^C]lactate (**B**) acquired using the metabolite-specific EPI sequence. The blue squares indicate the location of the kidney and the red squares the liver.

**Figure 6:**
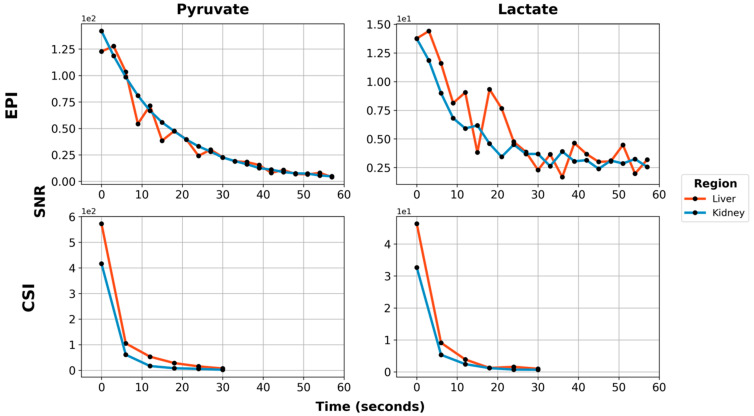
Dynamic metabolite curves for [1-^13^C]pyruvate (1st column) and [1-^13^C]lactate (2nd column), acquired using metabolite-specific EPI (1st row) or CSI (2nd row). Red lines are the hyperpolarized signal from the liver, while blue lines are from the kidney. Signals are shown in approximate SNR units. Note that the SNR scale is different for each subplot.

Using EPI with metabolite-specific SSRF excitation pulses provided an improved spatial resolution as compared with CSI (5 mm vs. 10 mm in-plane) and also allowed us to better characterize the temporal dynamics of the HP magnetization (Figure 5 and Figure 6). The temporal resolution obtained using EPI was 3 s and could even have been much shorter, whereas it was 6 s with CSI. Furthermore, despite the additional time points, EPI preserved the HP magnetization for a significantly longer amount of time. Although the early EPI frames contain the majority of the signal, we were able to detect both HP pyruvate and lactate signals 30 s from the start of imaging (Figure 5 and Figure 6). Conversely, with CSI, there was little to no HP signal remaining 20 s after the start of imaging (Figure 6). In addition, it is notable that CSI resulted in larger HP signals in the rat chest, most likely cardiac in origin.

**Figure 7:**
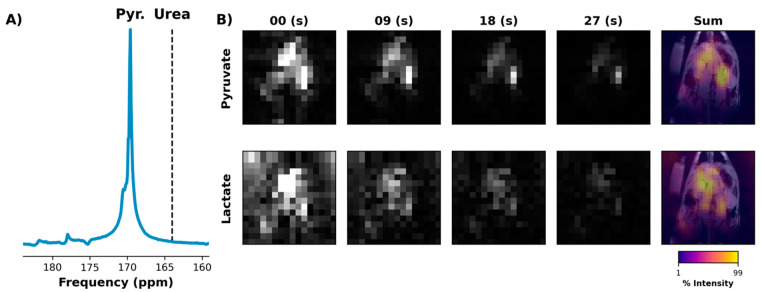
^13^C frequency spectrum acquired from 4 cm-thick slab that included the rat liver and kidneys but excluded the heart and lungs (**A**). The spectrum was obtained just after the injection of [1-^13^C]pyruvate using a small flip angle. An automated script was used to move the scanner center frequency from the urea resonance (dashed line) to that of pyruvate. The script then triggered the start of an EPI acquisition that used SSRF frequency offsets computed relative to the pyruvate resonance (**B**). Both pyruvate (1st row) and lactate images (2nd row) were of similar quality to prior experiments in which the frequency offsets were computed using a separate HP injection (Figure 4 and Figure 5).

For the improved EPI acquisition with fully automated center frequency calibration, an example initial 4 cm abdominal slab spectrum is shown in Figure 7A. Selected images from the EPI series that followed are shown in Figure 7B, showing a comparable SNR and signal lifetime to the prior EPI data in which CSI was used to confirm the necessary frequency offsets (Figure 5).

## 4. Discussion

We have demonstrated that EPI with SSRF pulses is an effective method for imaging HP compounds at 4.7 T. Both of our custom SSRF pulses were shown to selectively excite the resonance of interest (pyruvate or lactate) without exciting nearby metabolites. Subsequent in vivo imaging showed improved image quality and dynamics with EPI compared with conventional CSI acquisition. Finally, to minimize off-resonance effects, we developed an automated pre-scan procedure to determine and apply the correct pulse center frequencies based on real-time [1-^13^C]pyruvate data.

The fact that single-shot EPI acquires an entire image with a single RF excitation makes it an excellent fit for imaging HP compounds. Most HP agents have T_1_ constants of a minute or less [24], with T_1_ decreasing as the field strength increases above ~1 tesla [25]. Therefore, a prerequisite for any HP imaging method is the ability to acquire images quickly. It is also potentially advantageous to use as few RF excitations as possible, as each pulse consumes the non-renewable polarization. EPI satisfies both of these criteria, greatly enhancing our ability to measure signal dynamics in vivo. Pyruvate and lactate signals were both detectable 30 s after the start of imaging with EPI. By contrast, after 30 s, essentially no signal was detected with CSI (which requires 64 excitations per image, as configured). Furthermore, our EPI images were acquired with twice the temporal resolution vs. our CSI acquisition and could, if necessary, be collected much more quickly.

A large fraction of the performance improvement with EPI was probably due to the ability of our SSRF pulses to independently control the flip angle for individual metabolites. This allowed us to use a larger flip angle on lactate (increasing the SNR) while, at the same time, using a smaller flip angle on pyruvate (preserving magnetization for subsequent excitations). It should be noted, however, that the use of different flip angles between metabolites, along with uncertainties regarding the polarization level at the time of injection and rates of depolarization within different local microenvironments, prevented us from obtaining quantitative estimates of metabolite concentrations.

Although this is the first report using HP ^13^C EPI with SSRF pulses at 4.7 T, several investigators have previously used similar techniques at other field strengths. One of the first reports was by Cunningham et al., who used flyback EPI along with an SSRF pulse to measure lactate formed after injecting [1-^13^C]pyruvate at 3 T [15]. A subsequent study by Miller et al. developed SSRF pulses for lactate, pyruvate, and bicarbonate for use with flyback EPI at 7.0 T [9]. Other investigators have designed SSRF pulses for use with symmetric EPI or spiral readout trajectories [17,26]. Of particular interest is the symmetric EPI method used by Gordon et al. [26]. Whereas the flyback EPI proposed here only acquires data during the negative lobes of the readout gradient, symmetric EPI uses both the positive and negatives lobes during readout. This can result in an increase in efficiency, and thus in the SNR. Using a ^13^C phantom, Gordon et al. reported up to a 2× increase in the SNR with symmetric EPI over flyback EPI [26]. However, in the symmetric EPI approach, differences in odd/even k-space lines can introduce Nyquist ghosting artifacts.

The primary limitation of SSRF excitation for HP ^13^C MRI is that the resonance frequency for each metabolite must be known with high precision prior to acquiring data. If an incorrect frequency offset is used, the desired flip angle may not be achieved, and the pulse may excite other parts of the spectrum instead of the intended resonance. Furthermore, the resulting signal may be shifted in space as the spectral location of each metabolite must be known to correctly reconstruct the data.

One simple method for properly setting the frequency offsets is to express them relative to a known reference. For example, Cunningham et al. and Lau et al. used external lactate [15] and urea [17] phantoms, respectively. In this case, if the reference is not the same chemical species as the metabolite of interest, the frequency difference between the two resonances must be assumed. This is only a very minor weakness, however, as the chemical shift between individual metabolites at the same spatial location is generally highly consistent. However, it is not guaranteed that the resonance frequency of the reference is the same in the tissue of interest as it is in the phantom due to variations in the B_0_ field. B_0_ variations can be accounted for, however, through the use of a B_0_ field map [9].

We chose an alternative approach in which a single ^13^C spectrum is acquired over the region of interest after injection of [1-^13^C]pyruvate, but prior to EPI acquisition. This real-time scan feedback was used to adjust the pulse center frequencies automatically, thereby mitigating the effects of frequency shifts caused by B_0_ variations and eliminating the need to know the exact resonance frequency of [1-^13^C]pyruvate a priori. We found that this approach compares favorably with using a separate CSI acquisition to measure the in vivo frequencies for each metabolite, which is far more cumbersome. Despite these advantages, relatively few investigators have previously utilized such an approach [27,28,29]. This is probably mainly because most scanner environments cannot easily be programmed to adapt to real-time imaging feedback. The VNMRJ scanner environment is particularly amenable to such customization, facilitating our approach. However, a limitation of our strategy is it only measures the appropriate frequency offset for [1-^13^C]pyruvate, again applying prior knowledge of the spectral separation between pyruvate and lactate. However, as noted above, this is only a minor weakness as these spacings are generally consistent over a small spatial region. While, in principle, it is possible to also use the pre-scan spectrum to identify the location of the lactate resonance, we chose not to attempt this as the lactate peak is much harder to identify at this early time point due to its low concentration, particularly with the small flip angle necessary to preserve polarization for the subsequent EPI scan.

## 5. Conclusions

We have demonstrated that SSRF pulses can be used along with flyback EPI to quickly and efficiently image HP [1-^13^C]pyruvate and [1-^13^C]lactate at 4.7 T. Using EPI resulted in improvements in the signal lifetime and temporal resolution over a conventional CSI approach, without sacrificing the spatial resolution or image quality. We also developed an automated pre-scan procedure to make our sequence more robust to off-resonance effects. Taken together, these tools substantially improved the quality of HP imaging in our laboratory. We have made our custom SSRF pulses and source code (SSRF profile sequence and automated pre-scan procedure) available online so that other investigators can use these methods as well.

## Data Availability

The pulse waveforms, profile sequence, and automated center frequency script can be found online at https://github.com/cvonmorze/SSRFprofileAgilent (accessed 15 May 2021). All other data are available upon request from the corresponding author.

## Supplementary Materials

The following are available online at https://www.mdpi.com/article/10.3390/tomography7030040/s1, Figure S1: Dynamic ^13^C spectra from the liver and kidney.
