# Supplementary material for: Metabolite-Specific Echo-Planar Imaging of Hyperpolarized [1-13C]Pyruvate at 4.7 T
Source: Tomography. 2021 Sep 15;7(3):466–76. doi: 10.3390/tomography7030040 (PMC8482109; doi:10.3390/tomography7030040)
Supplement: Supplementary file 1 [file tomography-07-00040-s001.zip › tomography-1236112-supplementary.pdf]

Supplemental Material for Blazey et al., 2021

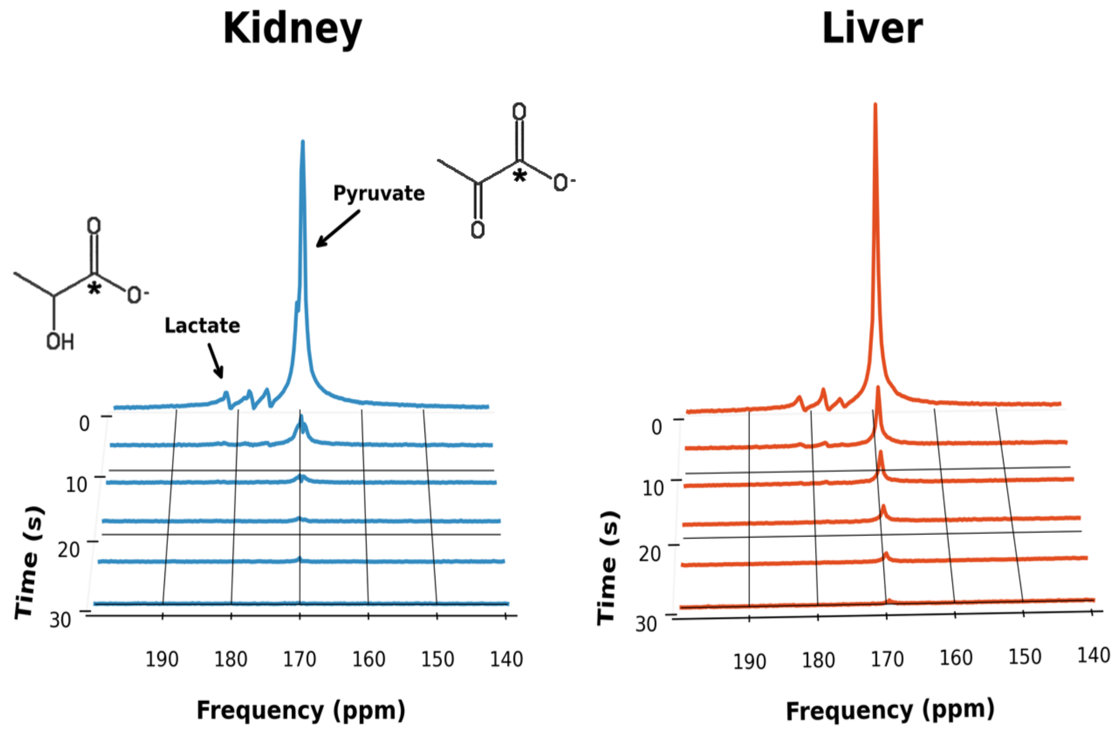

Figure S1: Dynamic ^{13}C spectra from the liver and kidney. All spectra are shown with the scale. Locations of each ROI are shown in **Figure 5**. ^{13}C-labeled positions are indicated by stars. All spectra were acquired using CSI as described in the main text.
